# Delayed diagnosis of pemphigus vulgaris in rural Nepal due to healthcare inaccessibility and harmful traditional practices: A case report

**DOI:** 10.1002/ccr3.8754

**Published:** 2024-04-11

**Authors:** Amisha Adhikari, Jameel Akhtar Mikrani, Samata Nepal, Ashish Rauniyar, Manish Chaudhary, Alok Atreya

**Affiliations:** ^1^ Lumbini Medical College Palpa Nepal; ^2^ Department of Dermatology and Venerology Lumbini Medical College Palpa Nepal; ^3^ Department of Community Medicine Lumbini Medical College Palpa Nepal; ^4^ Department of Forensic Medicine Lumbini Medical College Palpa Nepal

**Keywords:** awareness campaigns, healthcare accessibility, pemphigus vulgaris, rural Nepal, traditional remedies

## Abstract

Early intervention is imperative for potentially fatal dermatologic diseases such as pemphigus vulgaris. In rural Nepal, limited public awareness, home remedies, and delays in healthcare access lead to poor outcomes. Although biopsy confirms the diagnosis, experienced dermatologists can make an accurate clinical diagnosis when characteristic skin lesions are present.

## 1 INTRODUCTION

Pemphigus vulgaris (PV) is a rare autoimmune dermatological disease that results in blistering and erosion of mucocutaneous membranes due to cell death.^1^ Globally, the incidence of PV was 2.83 per million person‐years (95% CI, 2.14–3.61), with a consistent and stable occurrence between men and women for the past half‐century.^2^ The highest incidence rate of PV was in the Southern Asia subcontinents from multiple studies with an incidence rate of 4.94 per million person‐years (95% CI, 2.55–8.10).^2^ A 10‐year audit conducted in a tertiary hospital in Nepal reported that only 31 patients out of 710 dermatology admissions were PV.^3^ In nearly 80% of instances, the disease first presents itself with blisters affecting the oral mucosa before spreading to other parts of the body, sparing the palms and soles.^4^ The blisters rupture easily and form raw erosions with a red base, known as the positive Nikolsky's sign.^5^ Although the development of corticosteroids and steroid‐sparing treatments has improved the prognosis of PV over time, some patients still die of the disease due to infections and treatment complications.^1^, ^4^ The exact etiology of PV is still unknown. There are reports where PV is triggered by pregnancy.^6^ There are also instances where maternal antibodies permeate the placental barrier resulting in neonatal pemphigus.^7^ PV has infrequent presentations such as epigastric pain radiating to the neck caused by bleeding esophageal ulcer in the absence of a typical oral lesion.^8^ In one rare case, a female patient presented with ulcerated plaques on her foot that did not seem to heal.^9^ Patients are susceptible to extensive keloid scarring after a severe flare of the PV and superinfection.^10^ Full‐blown PV may be prevented by early intervention at the point of oral eruptions or skin lesions.^1^, ^4^

## 2 CASE HISTORY

A 36‐year‐old male attended the emergency department (ED) of a tertiary care hospital with a chief complaint of multiple fluid‐filled blisters all over his body, which easily ruptured and ulcerated, and that have been persistent for more than a month. According to his history, the first blister appeared in his mouth, causing dysphagia, before extensively spreading to his entire body. He gave a past medical history of a chronic dermatological condition persisting for 4–5 years, characterized by the presence of persistent silvery, scaly, and pruritic skin lesions predominantly on the head; which he had initially thought of dandruff and used anti‐dandruff shampoo as a home remedy, but his condition did not improve. He then visited multiple pharmacies and sought various treatments without experiencing significant improvement over the years. Despite these efforts, the condition persisted without achieving resolution of symptoms.

### 2.1 Examination

**FIGURE 1:**
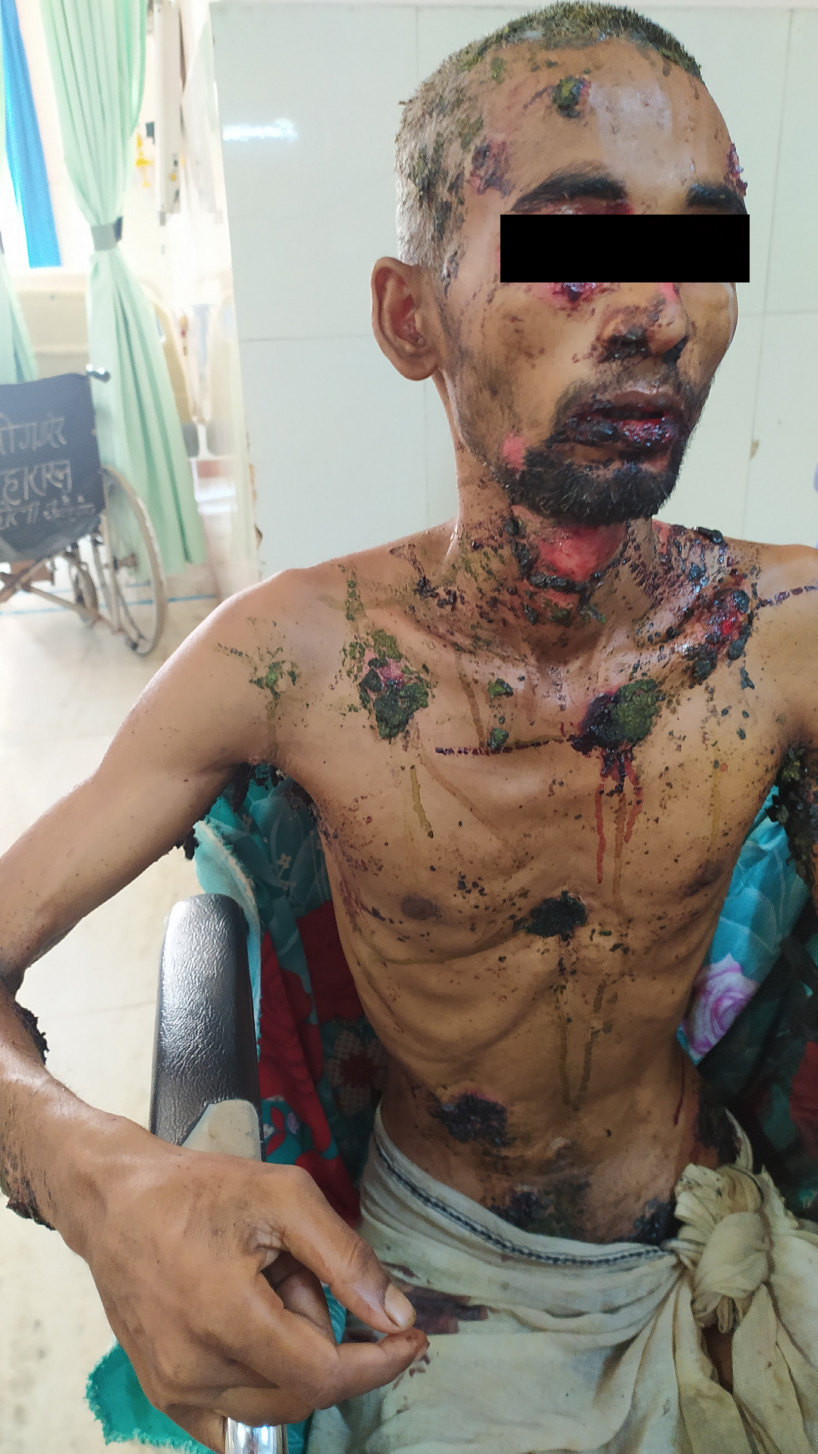
At presentation to the emergency room, the patient had most of the lesions smeared with a foul‐smelling greenish‐brown paste.

On examination, he appeared cachectic. The vital signs were within normal limits. There were multiple ruptured blisters from the scalp down to his lower limbs. They were widespread, some of which were on his face, neck, chest, arms and axilla, back, groin, and genitals. Most of them were smeared with a greenish‐brown paste, some were bleeding, and others had a putrid pustular discharge (Figure 1). Some unruptured blisters showed a positive Nikolsky's sign. When asked about the greenish‐brown paste, the patient revealed that he had been treating his wounds for the past 9 days, with cow dung, urine, and local herbal plants, which had worsened his condition.

### 2.2 Differential diagnosis and investigation

Considering the clinical presentation and positive Nikolsky's sign, we considered several differential diagnoses for our patient. The patient's state was consistent with pemphigus variant (pemphigus vulgaris, pemphigus vegetans, and pemphigus foliaceus) and, to some extent, with Stevens–Johnson syndrome and staphylococcal scalded skin syndrome (SSSS). The blood report showed moderately increased erythrocyte sedimentation rate (ESR) and mild anemia. No significant changes in total leukocyte count, prothrombin time (PT)/INR, liver and renal function tests, or random blood sugar levels were observed in the blood samples. The patient refused to undergo a biopsy. SSSS is commonly seen in children rather than in adults. The patient denied using any medications recently, and the skin lesions were not typical target ring‐shaped, which led us to exclude Stevens–Johnson syndrome. In the absence of the biopsy, the provisional diagnosis of PV was made based on the clinical presentation of the patient, including oral mucosal involvement before skin lesions, flaccid blisters showing positive Nikolsky's sign, rapid progression, and lack of healing tendencies. Biopsy is required for confirmation; however, PV can be accurately diagnosed clinically based upon the history and clinical features.

### 2.3 Treatment

The patient was thoroughly washed with soap and water to remove all the contaminants. He was then admitted and isolated in a clean room and treated with a regimen of dexamethasone, azathioprine, systemic antibiotics (Inj. Ceftriaxone 1 gm/bid for 10 days), regular paraffin gauze dressing, and local application of antibiotics twice a day. Based on the history, the patient likely suffered from psoriasis and his previous immunosuppressive therapy could potentially have influenced the onset and progression of PV in this case. He was married with two children and had worked as a wedge laborer in a metropolitan city in India before returning home due to the COVID‐19 pandemic lockdown. Currently, he was unemployed and engaged in agricultural work. Throughout the pandemic, he came across numerous social media posts advocating for alternative therapies. This influenced his choice to rely on home remedies instead of seeking medical care for his illness.

### 2.4 Outcome and follow‐up

**FIGURE 2:**
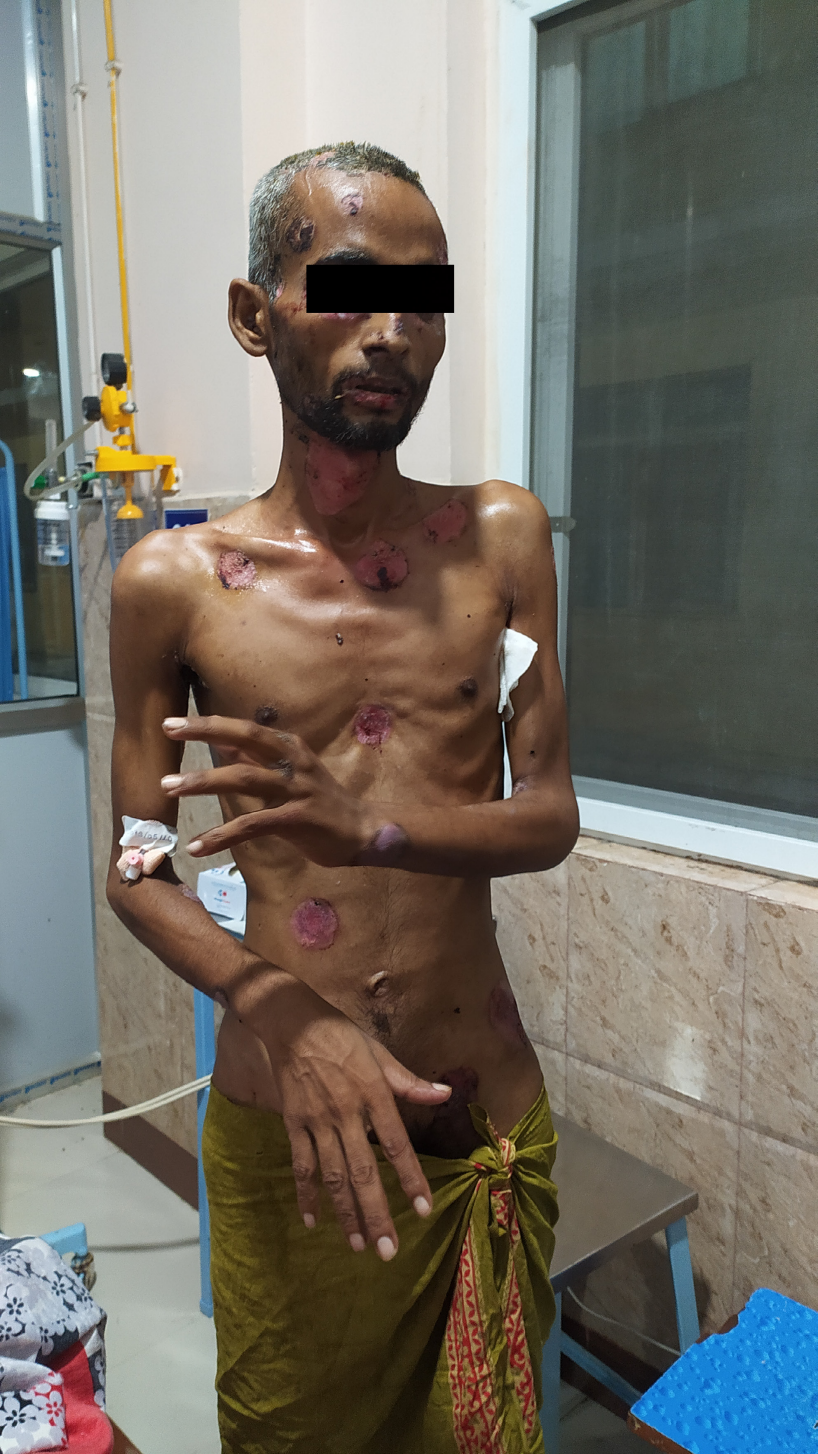
The lesions at the end of 2 weeks of treatment.

**FIGURE 3:**
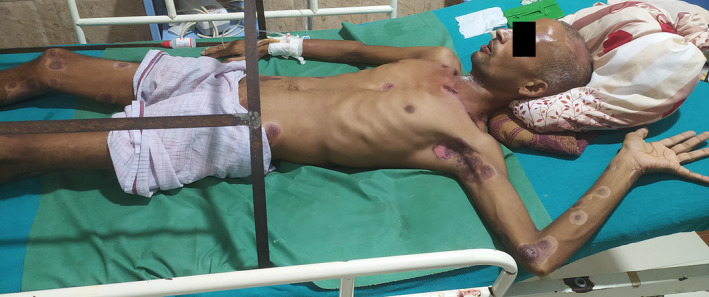
The wound at the end of 3 weeks of treatment.

The patient showed improvement in 2 weeks (Figure 2) with remarkable healing observed at 3 weeks (Figure 3) and was discharged after a month. The patient did not follow up thereafter.

## 3 DISCUSSION

PV is a relatively rare but life‐threatening autoimmune disease that disrupts adhesion between epidermal keratinocytes. This leads to blister formation, erosion, and poor wound healing.^1^, ^4^ In rural Nepal, the challenge of accessing medical care is compounded by the limited availability of tertiary care centers. The remote locations and rugged mountainous terrain make it arduous and time‐consuming to reach these vital facilities. This patient experienced financial hardship and physical suffering due to initially avoiding medical care. Earlier medical intervention may have better managed his condition, underscoring the need for public health awareness campaigns to educate rural communities on promptly seeking medical care for the symptoms concerned. Furthermore, in rural areas, many people have limited knowledge about maintaining good health and lack awareness of rare diseases such as PV. This lack of awareness can cause delays in seeking proper medical care, as the patient also had ignored his skin irritation, believing it to be a common benign condition initially.

The patient's recent unemployment, his return from abroad where he was working, and rural living may have contributed to emotional stress that acted as a PV trigger, as psychological stressors have been associated with PV onset in several reports.^11^, ^12^ His previous immunosuppressive therapy for psoriasis could have potentially influenced the onset and progression of PV in this case, especially given the known comorbidity between PV and other autoimmune conditions.^13^, ^14^ Viral infections may also have played a role, as studies have established that viruses like HSV can upregulate inflammatory cytokines and induce autoantibody production.^15^, ^16^ Finally, his rural environment and agricultural work may entail exposure to pesticides that have been linked to higher PV risk, likely by disrupting keratinocyte acetylcholine receptors.^17^, ^18^, ^19^

The patient's health decisions were influenced by the COVID‐19 pandemic and lockdown. With limited job prospects in his rural village, he started using social networks. However, unverified content that promoted alternative therapies involving, for example, cow dung, influenced his harmful reliance on such remedies. The absence of an authoritative body that could verify health information in Nepal allowed the spread of COVID‐19 misinformation, which influenced patients' decisions. It is also possible that the patient's cultural beliefs in traditional healing methods were targeted by local traditional healers, who made use of the lockdown restriction of the patient's access to mainstream healthcare. As a result, due to a lack of scientifically evaluated guidance, patients as in our case face substantial risks from misinformation or disinformation on unproven or potentially dangerous therapies.

This case highlights how limited rural health literacy and access to healthcare in Nepal delay the diagnosis and treatment of potentially fatal dermatologic diseases such as PV, causing worse outcomes.

A multifaceted approach will be necessary to improve the health literacy of rural populations in relation to rapid care of serious conditions such as PV. Awareness campaigns, enhancements of medical infrastructure and delivery systems, patient‐centered design of solutions as well as countering of health misinformation will be key. Partnerships between researchers, policymakers, healthcare providers, and the rural communities could facilitate the development of effective public health campaigns. Media platforms and telecommunication companies should only allow the dissemination of verified health information. Evaluation of traditional healing practices requires a nuanced scientific approach. All these measures will facilitate early diagnosis and evidence‐based management of dermatologic diseases.

Treatment was initiated based on the classical PV features observed in this case. While a biopsy confirms the diagnosis, it was unfeasible here due to the patient's refusal. Experienced dermatologists can accurately diagnose PV clinically in the appropriate setting, with characteristic oral blisters and skin lesions present.^20^ The risks of delaying treatment outweighed the uncertainty of the provisional clinical diagnosis. The goal of the treatment is to suppress the immune system with corticosteroids, often combined with steroid‐sparing agents such as azathioprine, mycophenolate mofetil, or rituximab.^1^ The goal is to quickly and effectively control disease and taper medications to the lowest effective doses to avoid recurrence. We used dexamethasone, azathioprine, and regular paraffin gauze dressing of the wound. In this case, we used broad spectrum antibiotics because our patient had applied herbal remedies to the wound, which may have contaminated it.

## 4 CONCLUSIONS

This case of PV in rural Nepal has demonstrated formidable barriers at the patient and system levels which hinder timely diagnosis and evidence‐based treatment of potentially fatal dermatologic diseases. These barriers include the lack of awareness, limited access to healthcare providers compounded with poverty, and the spread of misinformation which leads to harmful choices affecting one's health. Although overcoming these problems will be challenging and will require substantial efforts, public health strategies that facilitate health literacy, counter misconceptions, improve rural medical infrastructure and delivery, and enable patient‐centered care may be beneficial in achieving the target goals of preventing morbidity and mortality. Driving social and behavioral change among underserved groups will require the utilization of implementation research, collaboration with the community, and strong political commitment. These three factors will play a key role in bringing about the desired transformation. Overall, the main insight from this case is that multifaceted solutions facilitating early healthcare seeking are required to be patient‐ and population‐oriented as well as solutions to improve outcomes for life‐threatening conditions such as PV.

## AUTHOR CONTRIBUTIONS

**Amisha Adhikari:** Data curation; writing – original draft; writing – review and editing. **Jameel Akhtar Mikrani:** Data curation; validation; writing – review and editing. **Samata Nepal:** Writing – original draft; writing – review and editing. **Ashish Rauniyar:** Data curation; writing – review and editing. **Manish Chaudhary:** Data curation; writing – review and editing. **Alok Atreya:** Conceptualization; supervision; writing – original draft; writing – review and editing.

## FUNDING INFORMATION

None.

## CONFLICT OF INTEREST STATEMENT

The authors declare that they have no competing interests.

## CONSENT

Written informed consent was obtained from the patient to publish this report in accordance with the journal's patient consent policy.

## Data Availability

All data underlying the results are available as part of the article, and no additional source data are required.
